# Book Reviews

**Published:** 1822-08

**Authors:** 


### CRITICAL ANALYSIS
### OF
### ENGLISH AND FOREIGN LITERATURE
### RELATIVE TO THE VARIOUS BRANCHES OF
### Medical Science.

Quæ laudanda forent, et quæ culpanda, vicissim

Illa, prius, cretâ; mox hæc, carbone, *notamus.*—PERSIUS.

### DIVISION I.

### ENGLISH.

ART. I.—*Notes on the Medical Topography of the Interior of Ceylon, and on the Health of the Troops employed in the Kandyan Provinces, during the Years* 1815, 1816, 1817, 1818, 1819, *and* 1820; *with brief Remarks on the prevailing Diseases.* By HENRY MARSHALL, Surgeon to the Forces. 8vo. pp. 228. Burgess and Hill, London, 1821.*

THAT topography is a science intimately bearing upon medical pursuits, cannot be doubted; and it seems equally clear that no country calls more imperiously for its cultivation, on the part of the medical profession, than our own, whose colonial possessions are proverbial for insalubrity; yet details of locality and atmospherical phenomena are generally dismissed by the reader, as dry and unprofitable matter, unworthy of arresting his attention.

If any proof of the importance of these inquiries can be required, it may be afforded by the results of recent investigations on the nature of yellow fever, a disease which at length has been so clearly traced to putrid emanations from the soil, as to leave no reason for doubting that, in many spots where it now exerts its destructive ravages, it will be made to disappear by the removal of its cause, with as much certainty as intermittents and the plague have been banished from our metropolis.

The early chapters of Mr. MARSHALL's work are devoted to the medical topography of the interior of Ceylon, and its natural history; the character, habits, customs, and prevailing diseases of the indigenous inhabitants, are also considered; but we think it better to leave these particulars unnoticed, as the more important part of the work promises a supply of more materials than we can well include within a single Number of our Journal: we refer to that which relates to the prevailing disorders of our troops, including so much of the topography of the

* For this Review we are indebted to the author of the Retrospect published in the last Number.—EDIT.

country surrounding their garrisons as is necessary to elucidate the origin of disease among them.

The author informs us that the troops employed in Ceylon were Europeans, Caffries, Malays, and Indians; each class of which has particular diseases. Those which are described as more frequently affecting the European, are endemic fever, abscess of the liver, and dysentery. Why cholera is omitted we are at a loss to conceive, since, in another part of the work, it is said to have severely affected *all* classes of the troops, excepting only the commissioned officers. It may, however, be less frequent in its attacks than the other disorders.

"The Malay is liable to diseases of the chest, as consumption and asthma, but especially to pneumonia. He is susceptible, also, of endemic fever, which for the most part assumes the intermittent type; and scabies is a frequent affection among them. Their females are distinguished for fecundity; their children thrive, and contribute materially to recruit the regiment." We note the latter more particularly for the purpose of putting it in contrast with an opposite statement of the author respecting the Caffrie children, who, by the time they reach five or six years, droop, become meagre, and generally die before the age of puberty. For this high degree of mortality, the author professes himself unable to account. It seems that considerable pains have been taken to keep up the stock by the importation of a large proportion of females, who, with their children, were allowed regular rations. The offspring of indigenous females by a Caffrie father are as liable to disease as where both parents are Caffries.

Another very remarkable feature in the constitution of Caffries is their comparative insusceptibility to the cause of endemic fever, by which they rarely suffer even when the other classes largely experience its destructive influence. We are bound to presume upon the accuracy of the author's observation in this respect, but it appears to us paradoxical. The same race of beings in Madagascar, whence the troops in question are recruited, are liable to the inroads of pestilential fever, from which, as we remember to have learned from the Abbé Rochon, they secure themselves, in a certain degree, by remaining in their huts amidst a thick smoke. On turning to this writer's Voyage to Madagascar, we find the account sufficiently deserving of being transcribed, in connexion with the subject under review.

An adventurer, Benyowski, "chose the Bay of Antongil for the place of his principal establishment, though that part of the country is ravaged and desolated by pestilential fevers, from the month of October till the beginning of May. Navigators call that fatal season the winter. No doubt can be entertained that the noxious vapours which arise from the woods and marshes are the real cause of these epidemical diseases. The inflammatory air and putrid exhalations which proceed in abundance from water in a state of stagnation, and corrupted by the remains of vegetables, change the good quality of the atmospheric air during calm weather or great heats. On such occasions the air is seldom renewed by the sea breezes; the north winds carry these exhalations along the coasts, and drought and tranquillity render their effects more fatal. The Malegaches know, in a small degree, how to preserve themselves, by remaining in their huts or houses amidst a thick smoke; yet the most sober and robust of these islanders cannot always withstand the malignancy of the disorder. It is not surprising, therefore, that the Europeans who are obliged to reside on that coast should fall victims to distempers which attack even those who are seasoned to the climate."

Mr. Marshall, however, not only makes the general observation above mentioned, respecting their immunity from particular diseases, but in another part relates the fact, that, while "fever was making great ravages among the Europeans, a detachment of Caffries, consisting of about sixty individuals, continued healthy:" while, at the same time, the indigenous inhabitants, also, of the infected province suffered greatly.

The Africans in Ceylon are said also to be liable to cachectic disease and consumptions. Disorganizations of the lungs will take place to an incredible degree, without being suspected during life; the difficulty of detection in them might, however, the author suspects, be owing to their unintelligible language.

The Indian, we learn, is subject to scabies; to attacks of intermittent, where its causes prevail; and to inflammations of the lungs and intestines, under great transitions of temperature. He has but little fortitude under disease, and complains without appearing to have an adequate cause for his loud expression of suffering: "while his mind wants fortitude, his physical frame seems frequently to possess but a very moderate share of the principle of resistance to the inroads of disease, and of the powers of renovation. Life is often rapidly extinguished without much apparent disease. The mere pain of an irritable ulcer has sometimes appeared to occasion death."

At Minery, one of the stations of the troops, the endemic fever prevailed to a great degree, assuming a remittent or intermittent character, the type of which often varied in the same individual. Remittents were more common among Europeans than among other classes of troops. Sometimes the attack came on rapidly, and at others after several days of indisposition. The symptoms commenced with loss of appetite, listlessness, dorsal pains, chills alternating with heat; then ardent heat, head-ach, thirst, anxious breathing, white tongue, uneasiness in epigastrio, nausea, and in some instances vomiting. When the fever remitted, which commonly took place in the forenoon, about twenty-four hours after its accession, moisture came upon the skin. After a few hours the symptoms recurred; and the patient sometimes had several exacerbations and remissions in one day. As the disease advanced, the remissions were often scarcely perceptible; the tongue was covered with a brown or blackish fur; the skin became yellowish, clammy, and cold where it was exposed to the air; the pulse was small and quick. Nausea, vomiting, and eventually delirium, supervened, with hiccup, subsultus tendinum, and coma; and the patients died at different periods, from the fifth to the tenth day.

We are the more disposed to record this statement, since, as we have remarked in our Retrospect of the preceding month, a professed writer on Typhus has recently announced, as a clinical novelty, that intermittent and continued fevers would run into each other; a fact which, in situations where these disorders may be considered to be endemic, is as notorious as the noon-day sun.

Relapses were very frequent, especially at a station called Kotabawa. Under these circumstances, the patient often complains of dizziness and diminished power of locomotion, great restlessness, heat, quick pulse, thirst, delirium, vomiting, yellowness of the skin, severe pain of the thighs, legs, and feet. Syncope and diarrhœa were the immediate precursors of death.

Anasarcous appearances occurred here more frequently, and sooner after the accession of fever, than in other districts. The blistered parts were also observed to become sloughy.

On examination after death, no morbid appearances were found *where the disease terminated rapidly*; which leads the author to believe that the changes in the structure of organs, which were occasionally remarked, were the *consequence*, rather than the cause, of the symptoms. From the detail of these appearances under the heads of the several organs examined, we do not discover any decided marks of inflammation, but rather of vascular turgescence, and consequent effusion of serum. The pia mater, the lungs, the liver, and the spleen, were for the most part turgid with blood; the latter was sometimes unusually large. Serous effusion existed in the ventricles and between the membranes of the brain, often without any symptoms during life by which it could be suspected. It often occurred also in dysentery, where no affection of the brain was manifest: on the contrary, when coma, and other symptoms *usually considered* to denote oppressed brain, were present during life, no effusion was found; nor were the membranes and structure of the brain uncommonly vascular.

The villous coat of the large intestines was occasionally found dark, red, and pulpy, and sometimes with ulceration. The bile in the gall-bladder was sometimes found watery, at others brown or coffee-coloured; and often resembling pitch in colour and consistence.

Bleeding, purgatives, and cold ablution, were employed: "the relief the patients expressed after careful ablution, and particularly after the operation of a purgative, was often remarkable."

As the proportion of fatal cases is not here related, we turn back to another part of the work for information; from which we learn that, of 243 exposed to the climate of Villase, (whose principal post is Kotabawa, before mentioned,) between the 12th of July and the month of October, two only escaped fever; and the sum total of deaths was 193. On the 10th of September, thirty-three individuals joined the post at Kotabawa, from Trincomale: fifteen died before the 20th of October.

Twenty were sent from Trincomale to a position near the Minery Lake; not one escaped fever or dysentery: all were sent back sick to Trincomale. The mortality among them was great, but the precise degree was not known.

The 19th regiment had fifty-three individuals, of different ranks, exposed to the influence of the climate at Minery: they were all attacked with fever. Thirty-three died upon the road, or shortly after they reached the hospital. Fourteen survived the fever, but with greatly impaired constitutions.

One hundred and ninety-two healthy men passed *through* Vellase, early in October, to Oowa: towards the middle of the same month, many were seized with fever and dysentery, and twenty-nine had expired.

After the deplorable account of the mortality which prevailed in the provinces of Vellase and at Minery, the reader will naturally desire some particulars respecting the locality of these districts. Respecting Minery, the author observes, the post was remarkable for insalubrity: "It stood on the road leading from Trincomale to Kandy. The buildings constructed for the accommodation of the troops were placed in the immediate vicinity of a large artificial tank, generally known by the name of the Minery Lake. This extensive lake is situated in the flat country which stretches from the mountains of the interior to the sea. The wind from the south-west, or, as it is called, the Land-wind, blows very strong at Minery during the months of May, June, and July. The post was occupied in February, and it continued healthy until about the end of April. The months of February and March were wet, during which not a case of fever occurred; when the dry weather set in, fevers became prevalent. There being no medical officer at Minery, the sick were transferred to Trincomale, a distance of about sixty miles. On account of the violence of the prevailing disease, the distance from the hospital, and the imperfect means of conveyance, the sick reached Trincomale in a very deplorable condition. The insalubrity of the post not appearing to abate, the men were withdrawn about the end of June, and the position abandoned."

The greatest mortality appears to have been experienced in the flats of Vellase; yet, in this very province, Kotabawa was selected as an hospital station for the reception of the sick from the dependent posts. "This was one of a line of dependent posts that connected Balticaloe with Badula, leading through a low flat country that extends, without interruption, from the high hills which form the eastern rampart of the interior terrace, to the sea."—"No remarkable degree of sickness occurred among the troops on this line previously to the 10th of July. The month of June had been excessively hot, and the air dry. A hot wind blew from the north, which parched and withered vegetable life." It was from this time that the fever prevailed with the destructive consequences already related.

The mortality in Oowa was less than in the other districts; but the troops are described as having only *passed through* an unhealthy country. To pursue this inquiry, therefore, it is proper that we should obtain some information respecting the district of Oowa, which was their ultimate destination. We might, indeed, lay something to the account of the difference of season; but, as we shall lose nothing by an increase of our topographical acquirements, we shall retrace the ground which we had previously passed over. We learn that "fever became prevalent among the detachments in Lower Oowa towards the end of March; and, during the succeeding month, the troops employed in the province of Walapore became almost universally sufferers, either from fever or dysentery. Several of the posts of this district were abandoned in the month of May, on account of the sickness of the troops. Still, however, many insalubrious posts were occupied, and fever and dysentery prevailed to a very great degree. These diseases appeared among the troops that occupied posts formerly deemed healthy."

Having now ascertained that Oowa was also famed for its baleful influence over the constitution of its inhabitants, we travelled once more over the book for some tolerable account of the localities of the posts in this district.

Oowa is loosely described, in the beginning of the work, as possessing hills with tabular summits, intersected with winding patches of low ground, for the most part marshy, and very narrow. The author adds, "However deep the hollow may be, there is always an outlet for the water which may fall into it. There are, therefore, no natural lakes in the upper country.

The mountains, hills, and even the gentle swells, are in general covered, from their bases to their summits, with trees and low underwood. Indeed, the entire face of the country is nearly overspread with jungle and trees, by which means it has a woody impenetrable appearance. The hills in Oawa are, for the most part, exceptions to this observation. The mountains are covered with a coarse rank grass, and are in general almost destitute of trees."

Here we have all sorts of localities; but, whether the troops were posted in hill or dale, or in the midst of marsh or jungle, we cannot discover. The author speaks, indeed, of fever and dysentery occurring "in the interior *terrace*;" and, further, that the observations respecting the insalubrity of Oawa "are intended principally to apply to the prevalence of disease among the troops upon the hills;" from which we presume the most elevated spots must have been chosen for their location.

To what, then, was the insalubrity of the station owing? We would venture to suggest, that, although marsh and jungle are prolific sources of disease and death, *uncultivated land* has also a considerable share in the production of the same disorders. The "coarse rank grass," which never knew the mower's scythe, and which arrives at maturity only to fall again into a state of decay, affords copious materials, by its decomposition, for the production of disease. The author observes, that "the immediate neighbourhood of the large garrisons is as completely overrun with underwood and large trees as that of the smaller posts. In these extensive forests there is a quick reproduction of plants, and, consequently, a constant and rapid decay of vegetable matter." Again: In the close dells, where there is but little ventilation, *the decaying twigs and leaves emit a very offensive vapour.* It is not, however, to the soil alone to which we should look for the cause of disease in every case; and the author is of the same opinion. "On the interior terrace," he says, "I think we may, in a very great degree, attribute the prevalence of fever and dysentery to extreme fatigue, to great and sudden transitions of temperature, and varieties of weather; the men being often long under the influence of the sun by day, and much exposed to the *cold chilly dews of night*,—to frequently walking and sleeping in wet clothes,—to the scantiness and bad qualities of provisions,—to frequent long fasting,—depressing moral impressions,—and to great privations of the ordinary comforts belonging to the condition of the soldier."

Each of these circumstances is calculated to assist in the production of fever; but not one of the whole catalogue should be more guarded against than the night air of an insalubrious climate. It is in the night that contagion begins to stalk abroad.

The most accurate observers are unanimous on this point. Medical men, who visit their patients under yellow fever with impunity during the day, cannot venture out with safety after sun-set. It has been particularly noted that the seamen of ships in the harbour of an infected spot often disembark, during the day, in the midst of pestilence, with little risk; whereas the same individuals, having been exposed but a short time to the night air, have quickly fallen victims to the prevailing fever. Nor is the cause difficult to be explained. During the day, the *materies morbi* finds a rapid passage from earth to heaven, in consequence of the extreme rarefaction of air; but, "soon as the evening shades prevail," the pestilential miasma hovers about the soil which gives it birth; so that if, by an unlucky fatality, a man should either sleep or pass his night in the open air, he is literally, for that space of time, immersed in a vapour-bath composed of the most noxious materials.

From the following quotation it appears that, in the author's opinion, the origin of fever is not always to be traced to the soil.

"Remittent fever frequently occurs in all sultry climates, particularly if they are uncleared, and overrun with forests or low brushwood. The neighbourhood of extensive swamps, or half-inundated flats, is found to be often highly pestilential. In every part of Ceylon the remote cause of fever is therefore abundant. ***But fever is not always found to prevail in proportion to the apparent exuberance of the phenomena which are supposed to occasion it:*** sometimes fever is very general among the inhabitants in particular tracts of country, where the causes which are supposed to produce it do not appear to exist in any remarkable degree. Both dysentery and fever are occasionally found to prevail in particular stations on the coast, where marsh miasma do not appear to be abundant, and where, from the vicinity to the ocean, it might be supposed that the atmosphere of the land, if unhealthy, would be rendered much less so by the breezes from the sea. Hence, in as far as regards the remote origin of fever, it is necessary to speak in very general terms."

The author, in speaking of the relief afforded by ablution and bleeding, remarks that "frequently, although the symptoms were meliorated, a fatal issue could not be averted." This is precisely such as might be expected, in another case, from an attempt to cure a man labouring under symptoms from the poison of arsenic, while successive doses continued to be administered. As endemic fever is the effect of poisonous miasmata, the first indication of cure is to remove the cause, or to convey the patient from the source of infection. Recoveries, indeed, are occasionally effected while the system is still exposed to the noxious climate; but we think that, in these cases, more is due to a certain degree of resistance on the part of the constitution, than to the bleeding, purging, and cold ablution, of the practitioner.

Wherever, then, the nature of military service unfortunately requires troops to be stationed in the midst of marsh and jungle, healthier spots should, *if possible*, be selected, and suitable buildings erected for the reception of the sick, who should be transported thither as soon as the precursory symptoms of disease appear.

In answer to this it may be urged that, in districts remarkable for insalubrity to a considerable extent, no healthy spot can be found: but cannot the most unhealthy be converted into one of comparative salubrity, by the destruction of jungle and the draining of marsh and low lands, or the perfect cultivation of all? Much also, we are persuaded, could be effected by the erection of hospitals, whose structure should protect its inmates from the withering influence of particular winds, or from the scorching rays of the sun. An attention to details of this description would contribute more to the preservation of health in tropical climates, than the whole materia medica combined.

Prevention should be the first study of the practitioner: when this is impracticable, the removal of the patient from the *fomes morbi* becomes the paramount consideration.

We were struck with the inconvenient mode of conveying the men who dropped upon a march, which was adopted in the author's campaign; to swing the patient in a blanket upon a bamboo between two Coolies, when, under almost any circumstances, a more convenient conveyance might have been contrived upon the spot, argues, we think, a culpable omission some where. That the surgeons attached to the respective regiments employed are not chargeable with this neglect, we have reason to believe; as the author informs us that unusual difficulties presented themselves even to the conveyance of commissariat stores.

*Inflammation of the liver* is described as more frequently affecting the European than the native soldier, but no classes are exempt from it. "Among Europeans, and particularly among new arrivals, the disease supervenes sometimes with well-marked symptoms, namely, considerable febrile excitement, urgent thirst, and violent pain in the region of the liver. The pain is generally increased by pressure. The following symptoms are frequently concomitants of diseased liver, but not always: vomiting; cough; impeded respiration; increased pain when lying on the left side; pain on the top of the right shoulder; diarrhœa; costiveness. In one instance, a patient suffering under inflammation of the liver was, for a period of several days, unable to move his right arm; the hand was at the same time swelled and painful. Sometimes, particularly among individuals who have been long assimilated to a hot climate, the symptoms which precede and accompany the formation of an abscess of the liver are very little noticed; not unfrequently they pass entirely unobserved. This circumstance is particularly remarkable when inflammation of the organ is combined with that of the villous coat of the large intestines, giving rise to dysenteric symptoms. The progress of inflammation of the liver is sometimes very rapid. In general, it terminates either by resolution or by the formation of an abscess in the parenchymatous substance. The degree of rapidity with which pus forms in the liver is not in proportion to the local uneasiness or vascular excitement of the system. There is much reason for supposing that an abscess sometimes forms in the liver as rapidly without local pain, as when the uneasiness is very great. Death occasionally happens before suppuration takes place."

The morbid appearances after previous disease of the liver are various. Sometimes the organ is turgid with blood, giving it a dark-red colour; the peritoneal coat is found unusually red "in some very few instances," when symptoms of inflammation of the liver had existed during life. The traces of inflammation through the whole organ are often not equally manifest.

Abscesses of various extent are frequently found; some not larger than peas; in one, the quantity measured ten pints. The contents of the abscesses are, in general, well-digested pus: "sometimes they contain a serous whey-coloured fluid, with flakes of tenacious lymph or purulent matter floating in it. In some cases, where two abscesses were found in the liver, I have seen one filled with pus, while the other contained a fluid resembling the serous and colourless part of milk. The lining of the serous abscess is smooth, and apparently organized. I have succeeded in peeling off the membranaceous substance, below which the liver was smooth and seemingly sound."

The contents of abscesses in the liver are sometimes expectorated through the lungs; and sometimes, though rarely, recoveries take place under these circumstances.

In one case, the abscess passed into the stomach, in consequence of adhesion between this viscus and the liver.

In three cases, the abscesses were punctured, and their contents evacuated: the largest contained about fourteen ounces of pus.

The liver was sometimes softer; in others the parenchymatous substance had undergone considerable change. In some the peritoneal coat was easily peeled from the glandular substance, which was frequently granulous, and had lost much of its natural tenacity: this state also accompanied dysentery.

Sometimes the gland was friable, and easily divided. In one case, the appearance was that of hasty-pudding.

Sometimes the liver was harder than natural, and had a gritty feel on being cut into it.

In drunkards, the livers were yellowish, and contained little blood.

Diseased liver, the author observes, is not easily distinguished when complicated with dysentery. "We know that frequent dejections of blood, mixed with mucus, almost invariably indicate an inflamed condition of the villous coat of the *large intestines,* and that dysentery is frequently combined with diseased liver. It is a remarkable circumstance that, when the large intestines are inflamed, the proofs of an excited state of the liver are very obscure." The former predominates so much that the latter passes unobserved. The liver and the arch of the colon are so contiguous, that, when pain is produced by pressure in this part, we are at a loss to know whether it originates in the colon or the liver.

Inflammation of the liver is considered by the author to occur more frequently in Ceylon than elsewhere. The principal causes are, "exposure to the direct rays of the sun, and fatigue in this situation; a variable atmosphere; the habitual use of spirituous liquors; intoxication, and the irregularities that accompany such a state of mental derangement."

On the treatment of inflamed or diseased liver, the author is very brief, and very far from being satisfactory. He speaks very slightly of mercury, upon which other practitioners so fully rely. Whether this arises from any doubt on his part respecting its efficacy, or from inadvertence, we cannot divine. The author recommends copious bleeding, and purgatives, *part of which* should consist of calomel: but we hear nothing of the prompt employment of mercurial frictions, which we are taught to believe are of paramount importance in hepatitis and other pyrexiæ of tropical climates. It would appear, from the following quotation, that the author confines the use of this remedy to the chronic stage.

"Should the acute symptoms subside without a return of health, and there be no manifest proofs of the formation of pus in the liver, the use of mercury may then be tried, with frequent moderate purgation. A mild degree of salivation is sometimes useful in this stage of the disease, when the liver contains an abscess. I suspect that no quantity of mercury will cause ptyalism. Under such circumstances, the exhibition of mercury frequently occasions a soreness and heat of the gums, but rarely, if ever, ptyalism."

ART. II.—*Medico-Chirurgical Transactions, published by the Medical and Chirurgical Society of London.* Vol. XII. Part I. 8vo. Longman and Co. London, 1822.

THE first part of a new volume of the Medico-Chirurgical Transactions having been published, we hasten to lay an account of it before our readers. In doing this, we purpose only to give such an analytic sketch as may convey a general idea of its contents; and which we hope may induce those within reach of it to make themselves masters of the volume itself, while it may serve to gratify the wishes of those not equally fortunate. To criticise each paper would be a tedious, perhaps rather an invidious, task: we shall, therefore, confine ourselves to such remarks as are naturally suggested by the perusal; and, before we begin, we have no hesitation in expressing the satisfaction we have derived,—the volume before us affording an excellent illustration of the interest that may be given to cases, by connecting them with judicious practical reflections. We are the more inclined to insist upon this, from having frequently remarked among medical men a disinclination to listen to the detail of individual cases, as if no benefit could be derived from them. The records of medicine are built of such materials; and the publication of cases affording any feature of novelty, particularly of a practical nature, is a duty which every practitioner owes to the profession of which he is a member. The Transactions of the Medico-Chirurgical Society are not on a scale sufficiently extensive to communicate to the public all the papers, the contents of which deserve to be recorded; and we trust that those who see the advantages of increasing our stock of *facts*, will place their observations on record through the medium of *other* Journals. One case we have omitted,—not from its being deficient in interest, but because it appeared in a former Number, and has been re-published by some singular inadvertency.

*Attempt to drink boiling Water.*—The first paper, with its appendix, contains an account of six cases of children who had attempted to swallow boiling water. The most remarkable circumstances connected with these is, that, in the five first, the symptoms were such as to indicate an affection of the respiration, similar to that which occurs in croup; and Dr. HALL thinks it probable that the water does not actually penetrate to the stomach, but is arrested by spasmodic action of the pharynx. All the six cases proved fatal: in one of Dr. Hall's, temporary relief was afforded by tracheotomy; and, in this instance, the examination after death showed the epiglottis to be in a swollen, blistered, and corrugated state, with a similar condition of the tongue, mouth, and fauces; which, however, did not extend to the œsophagus. In the sixth case, which occurred at Saint Bartholomew's Hospital, the respiration was but little affected, and the symptoms led to suspicion that the brain was the seat of disease. Slight effusion was accordingly found between the membranes, and into the ventricles. There was just sufficient redness and tumefaction about the larynx and pharynx to show that they had been the seat of irritation. The trachea, stomach, and œsophagus, were sound.

*Operation for an Aneurism of the Subclavian Artery.*—This operation was performed at the Winchester County Hospital, on the 19th of March, 1821, by Mr. Charles Mayo, upon a fisherman, aged thirty-eight. The tumor corresponded to the situation of the artery; the pulsation was very strong, yet the clavicle was but little elevated. A transverse incision was made about two and a half inches in length, along the upper edge of the clavicle, and from the middle of this another was made directly upwards along the edge of the sterno-cleido mastoideus muscle. The artery was exposed by careful dissection; but even more than the usual difficulty was found in passing any instrument under it, so firmly was the vessel attached to the rib. This was at length accomplished by means of Desault's elastic needle, and the ligature tightened by Mr. Ramsden's instruments for that purpose. The pulsation in the tumor ceased, but was again perceptible on the following day. Great restlessness came on, and symptoms requiring repeated bleeding and opiates. By the 25th, the pulsation in the tumor was nearly as great as before the operation, and some hemorrhage took place from the wound: this hemorrhage returned on the following morning; the pulsation ceased, and the tumor became flattened. The bleeding recurred several times, and the patient gradually sunk, and died on the 31st. The most remarkable circumstance on dissection was the state of the artery. It had been completely divided, and the portions separated about a quarter of an inch; the extremity next the sac was much contracted, and filled with coagulum; that next the heart was not at all contracted, and was open to its extremity, which was only in part filled by lymph, there being an aperture that admitted the end of a probe between the lymph and the extremity of the vessel. This want of coagulum appears to have been caused by several large arteries taking their rise only half an inch from the spot where the subclavian had been tied. Mr. Mayo is of opinion that the fatal result is to be attributed to the general constitutional derangement, rather than to the loss of blood.

*Case of successful Bronchotomy.*—In this case a boy, four years old, fell with several pebbles in his mouth, one of which was drawn into the rima glottidis, and would have caused speedy suffocation, but for a young lady, who, in attempting to remove the stone, forced it into the trachea. On this the child became tranquil, and the presence of the foreign body could only be perceived when coughing was excited; at which times there was frequent danger of suffocation, from the pebble being forced up to the glottis. Next day (July 20,) the stone was extracted, without difficulty, by the usual operation of trachecotomy: it was of the shape of a kidney-bean, $\frac{1}{2}$ an inch long, $\frac{3}{4}$ of an inch wide, and $\frac{1}{4}$ of an inch thick. Symptoms of inflammation of the lungs came on, which yielded, by the 24th, to bleeding and other remedies; by the 27th, air had ceased to pass by the wound, and from this time the child gradually recovered.

This case is followed by some observations from the pen of Mr. HENRY EARLE, pointing out the propriety of having recourse to the operation when foreign bodies are lodged in the trachea, and alluding to three recent cases, which proved fatal from the body being allowed to remain. It appears, from the experiments of M. FAVIER, that foreign bodies are always forced out when a few of the rings of the trachea are divided. The comparative quiescence of the symptoms when the body falls into the trachea, and their exacerbation when it is forced towards the glottis, illustrates well the comparative degrees of sensibility in these parts.

*Singular Variety of Urine.*—In this case, communicated by Dr. MARCET, a child in good health passed urine, which, although colourless when voided, soon afterwards became nearly black. One specimen remained unaltered at the end of seven years; others, however, became putrid at the end of a few days. Upon being subjected to analysis by Dr. PROUT, it was found to contain neither lithic acid nor urea. From his experiments he concludes that the black urine owes its colour to a compound of a peculiar principle with ammonia, and that the black principle itself may be regarded as a new body possessed of acid properties. Another instance is mentioned by Dr. Marcet of black urine, which occurred in a girl subject to daily attacks of a febrile and hysterical character. To these we may add a third, which occurred last autumn, in a patient who applied at the Westminster General Dispensary on account of general debility, with occasional pains across the loins. The urine was exactly like ink slightly diluted with water. He brought a small phial of it in his pocket at each visit, which was handed about among the pupils; and his convalescence was marked by the gradual return of the urine to its natural state. He took opium and purgatives, and was about a month under treatment.

*Extraction of a living Fetus from a Woman killed by Violence.*—A woman, aged thirty, was run over by a stage-coach, and carried to St. Thomas's Hospital, where she died, twenty minutes after the accident had happened. Thirteen minutes after her death, the Cæsarean section was performed by Mr. GREEN, and a fœtus extracted, which exhibited no signs of life. A tracheal pipe was introduced, and, after artificial respiration had been carried on for a quarter of an hour, the child began to breathe; at the end of five minutes, three complete natural respirations were performed in each minute. A small quantity of brandy was now given: "this, however, evidently produced an injurious effect, for the breathing became weaker and less regular." We confess that, as the case is related, we cannot perceive what indication authorized the use of this strong stimulant: breathing had begun, and was evidently going on favourably, for, "with some assistance, the lost ground was regained, and in another five minutes the respiration might be considered regular and natural." The next measure was to put the child into *warm* water; and, as this deranged the pulse and breathing, it was removed, and, after it had somewhat recovered itself, was "just dipt into *cold* water," which is said to have produced no marked effect. We are told, in the preceding paragraph, that the infant, although at first apparently dead, had been recovered by means of artificial respiration of the lungs, and that the respiration had become regular and natural; "at the same time, the pulse at the wrist could be distinctly felt." Now, under these circumstances, we are as much at a loss to discover the object intended by this alternate use of the warm and cold bath, as by the exhibition of the brandy. The efforts made to save the child (which was removed from the hospital the first night,) proved unavailing, and it died thirty-four hours after its removal from the uterus. Mr. Green is of opinion that the death of this child is to be attributed to "the want of proper care in maintaining the animal temperature, and supplying fit and sufficient nutriment; and *to* the obstruction to respiration produced by mucus in the trachea."

*Man who swallowed Clasp-knives.*—This case is related by Dr. MARCET, and is the same which was noticed in several Journals ten or twelve years ago, the patient having died in 1809. No such particular or authentic record of it, however, has been published as that now before us. It appears that this infatuated man swallowed, at different periods, not fewer than thirty-five knives; some of which he voided by stool, but others, remaining in the stomach, put a period to his life, although not for ten years after his first experiment. On examining the body after death, a great number of pieces of the handles and blades of knives were found in the stomach and bowels; some of them, particularly a blade made of cast steel, were in a state of great preservation; others, again, were very much eroded. Some portions were so situated as to render their expulsion from the body impossible: one of the back springs had transfixed the colon opposite the left kidney, and projected into the cavity of the abdomen; another stretched across the rectum, and had one of its extremities fixed in the muscular parietes of the pelvis. No fæces had escaped, owing to the parts adapting themselves very closely round the instrument. This paper is followed by a letter from Dr. LARA, giving some account of the man while on-board his ship; and by the history of the patient drawn up by himself, certainly not the least interesting part of the communication.

*Case of premature Puberty.*—The pudenda of this child were observed to be unusually large at his birth; he was covered with hair, particularly the back of the head, and his voice was hoarse. When he was four months old, hair began to grow on the pubis, which was soon covered; the penis increased in size, and soon after the expiration of a year he had nocturnal emissions. Mr. SOUTH, by whom the case is related, says, he "has a fine, high, and spacious forehead; but the occiput is extremely prominent, from the enormous size of the cerebellum, which Drs. GALL and SPURZHEIM state is always the case when the genital organs are developed in a great degree." How completely these are developed, will appear from the following measurement. It is to be observed that he was born Sept. 6, 1818, and the account is dated January 8th, 1822. Length of the penis, when pendent, 3 inches; when erect, 6 ditto; circumference, when pendent, $3\frac{1}{8}$ inches; when erect, 4 ditto. He weighs 64 lb. avoirdupois, and his height is 3 feet 7 inches.

*Products of acute Inflammation.*—The author of this paper objects to the use of the term "coagulable lymph;" and is of opinion that the substance thrown out during adhesive inflammation is fibrin in conjunction with serum. Fibrin may be distinguished from coagulated albumen thus:—fibrin exhibits a fibrous structure, albumen does not; fibrin is firm and elastic, resisting considerable pressure; albumen, when coagulated, is readily broken down into a pulpy form; lastly, fibrin becomes solid spontaneously, when taken from the living vessels; albumen does not. Mr. DOWLER (the author of the paper,) thinks that fibrin is poured out in a fluid state, and, becoming solid soon after, incloses the serum betwixt its fibres: if this mass be pressed *strongly* in a fine linen cloth, fibrin may be obtained, exactly similar to that of the blood. The result of his experiments lead him to conclude, that the buffy coat which appears on blood drawn from persons labouring under acute inflammation, is not altered fibrin, as M. DEYEUX thought; neither coagulated albumen; nor that it consists entirely of coagulable lymph; but that it is composed of a tissue of fibrin, containing a large proportion of fluid serum between its fibres. This paper is followed by a note from Dr. Bostock, alluding to some experiments of his in 1807–8, the results of which led him to suppose that the buffy coat was principally composed of fibrin: at the same time he doubted whether it was exactly similar to the fibrin of the blood, and thought that a certain quantity of coagulated albumen was united with it.

*Use of Cubebs in Gonorrhœa.*—This paper is from the pen of Mr. Broughton, and the results of his experience are favourable to the cubebs; as will best appear from the following extract:

"Among fifty cases treated with cubebs, there were, therefore, three total failures, five relieved, and one suffered a relapse; leaving forty-five cases cured under the use of the pepper in less time than a month, with *one* exception; the largest proportion in less than three weeks, and several in a few days, among which latter some were well in eight-and-forty or thirty-six hours; and, failures excepted, the relief in all cases where the symptoms were urgent was very sudden; and only two instances of swelling of the testicles occurred, and one of chordee, continuing after the exhibition of the pepper, although, in other respects, the clap was relieved directly."

Mr. Broughton agrees with those who have gone before him in thinking that it should be discontinued if it does not act within three or four days. The preparations he employed were the powder, the wine, and the tincture; the first in doses of ʒss. to ʒij., and the others from ʒj. to ℥ss. twice or three times a-day.

The next paper in the order of succession is one by Mr. Shaw, on *Partial Paralysis;* but, as it seems, to us, too important for the limited analysis we are now giving, we shall delay its consideration till another opportunity.

*On Uterine Hemorrhage.*—Dr. Gooch has met with cases in which alarming hemorrhage took place after delivery, although the uterus had contracted "in the degree which commonly indicates security;" and this, he conceives, may occur under two circumstances. The first of these is where the patient is more than usually affected by loss of blood,—where there is a proneness to syncope. Such a person will suffer to an alarming extent, although the loss of blood be comparatively small; and of this Dr. Gooch relates two instances. The second circumstance is when the uterus contracts as usual, but the force of the circulation, being unusually great, overcomes the disposition in the mouths of the vessels of the uterus to contract, and so blood bursts forth. When this happens, it is to be stopped in the usual way, by the introduction of the hand and the application of cold. Dr. Gooch has succeeded in making the uterus contract by pouring cold water upon the abdomen from the height of several feet, where pounded ice had failed to produce this effect.

*Observations on Compound Fractures.*—This is a very sensible paper by Mr. Dunn, of Scarborough, giving an account of two cases of compound fracture, in which the projecting extremities of the bone were amputated with the best effect. In one of these, the portions of the tibia thus removed included three inches of the whole cylinder of the bone, yet the boy recovered so as to walk without crutches, the limb being only one inch shorter than the other. The second case is less remarkable, as a smaller portion of bone was removed: its termination was equally favourable. A third case is related, in which the bones in a simple fracture united in such a manner as to leave a sharp projecting edge of the tibia, which irritated the skin. A semi-lunar incision was made, the integuments reflected, and the angular portion of bone removed: the wound healed in four days.

*Umbilical Hemorrhage.*—The subject of this case was a boy, in whom the funis separated on the sixth day, without any remarkable circumstance. On the eighth it began to bleed, and continued to do so (notwithstanding the use of plugs and application of pressure,) for twenty-seven hours; at the end of which time the child died. On dissection, the umbilical vein was found filled with fluid blood, and was nearly as large as a goose-quill; both the umbilical arteries were sufficiently pervious to admit a probe. Mr. Pont, who relates the case, suggests the propriety of cutting down upon the arteries, and tying them, should such another instance occur.

*Vaccine Disease and Measles.*—A case is related by Mr. Gilder, assistant-surgeon to the Coldstream Guards, of a child, in whom vaccination was performed while it was under the influence of the infectious effluvia of measles. Both diseases run their usual course simultaneously, without modifying each other.

*Ununited Fracture of the Humerus.*—Mr. Earl justly remarks, that the records of medicine are too generally filled with the accounts of successful practice, while the unfortunate cases are either slightly alluded to or entirely omitted. Nothing certainly can tend more directly to retard the progress of a science, depending so much upon experiment and fact, as this disingenuous line of conduct; and the profession are, therefore, indebted to Mr. Earl for laying before them the result of his experiments, although they have proved unsuccessful. There is one sentiment, however, expressed by the author, in which we cannot concur. He says, "Although in these instances the practice was not crowned with success, I do not consider that the failure can be attributed to the operation, or that it at all militates against the propriety of adopting it in other cases."—Now, it is quite obvious that the history of an operation which has failed every time it has been tried, at least says nothing in its favour; and, having been tried, without success, under the direction of Mr. Earle, Mr. Brodie, and Mr. Green, militates, in our opinion, very strongly against the adoption of this operation in *similar* cases. A gentleman had the left arm fractured, and nothing remarkable occurred till the removal of the splints, when it was perceived that no union had taken place. About eight months after the accident, he placed himself under the care of Mr. Earle: at this time the broken ends of the bone were moveable upon each other, and were not in close coaptation. A seton was passed between them. Considerable inflammation and discharge followed; thickening of the parts about the fracture took place, and hopes were entertained of a favourable result. These, however, were disappointed; and, at the end of seven weeks, the seton was withdrawn, "having totally failed in producing any bony deposit." Mr. Green suggested the application of caustic potash to the ends of the bone; and this was accordingly done, after they had been laid bare by means of a free incision over the fractured part. Inflammation and thickening of the neighbouring parts followed; the patient felt the limb stronger; and sanguine hopes were again entertained. An apparatus was contrived so as to give steady support to the limb, which he wore for about three months: on removing it, the limb was as useless as before; any callus that might have been formed having been again re-absorbed. A similar experiment, and with a similar result, was tried in another case; but it is proper to remark, that both subjects were unhealthy.

*Large Nævus Maternus on the Head cured by tying the Carotid Artery.*—The title of this paper sets forth all it contains. The case is from Dr. Arenat, of St. Petersburgh, and runs as follows:

"A man, who had from his birth several nævi on different parts of his body, received a blow on one of them situated on the right temple. It increased rapidly in size, acquiring a prodigious bulk in the space of two hours after the injury. The carotid artery was tied, an inch and a half above the clavicle, and two ligatures were placed round it, half an inch distant from each other. The tumor burst during the operation, and the loss of blood was calculated at not less than eight pounds. On the day following, the tumor was found entirely emptied of blood: a great portion of the skin was now cut away, and about twelve small arteries secured. The ligatures on the carotid artery were removed on the seventeenth day, and the wounds healed rapidly afterwards."

We cannot too much commend the plain *business-like* way in which this short, but complete, account is detailed.

*Wounded Nerve of the Thumb.*—This case occurred in the practice of Mr. Wardrop. A gentleman cut his left thumb across the radial side of the distal phalanx: the wound was very painful, but healed readily. In a few days after, symptoms of general irritation came on, and by the end of three weeks he had lost flesh; the thumb, at all times painful, was particularly tender to the slightest touch; the pain extended to the arm, shoulder, and back. The nerve was divided by means of a deep transverse incision above the injury. This was followed by instant relief; but when, from any cause, the stomach is disordered, he still feels pain in the thumb. Some short observations follow on the effects of injuries of the nerves, recommending division above the seat of injury.

*Carcinoma Mammæ.*—This paper of Mr. CHARLES BELL'S has for its object to give a description of the true cancer of the breast, and to describe two or three varieties of disease to which that part is liable, and which are usually comprehended under the name of carcinoma. Mr. Bell commences his paper with a vivid and accurate description of the symptoms and progress of true cancer, which he says belongs to that term of life when the uterine functions cease: he notices the effect of age on the whole class of carcinomatous tumors; the same disease running its course rapidly, and with every symptom aggravated, in a woman of forty-five, while it will remain stationary for years in one of sixty or seventy. In combating the opinion that *tumor* is not an essential character of cancer, he observes, that, though that disease is often accompanied with a diminution in the bulk of the breast, that is not the case with the *tumor*, which is undoubtedly a preternatural growth, although the fat is sometimes absorbed and the gland shrunk, so that the whole mass is less than the natural breast. The absorption of the fat will also produce a difference in the external character, when there is none in the structure: in this case, the tuberculated irregularities will be apparent to the touch and eye; whereas, when the fat has not been absorbed, the whole breast will appear round, smooth, and full.

The varieties he describes are—1, *Impostumated Cancer.* This is, in fact, no more than a deceptive appearance in the progress of the disease, when, previous to ulceration, the breast appears to soften and point, as if an abscess was forming: this is only the prelude to the formation of a large, foul, and sloughy chasm. It is important to remark that, as the local disease makes its progress in two ways, by ulceration, and by mortification and sloughing of successive portions, great variation in the aspect of the sore is produced from day to day.

2. *Carcinoma Mammæ Hydatides.*—In applying this term, Mr. Bell is not influenced by Dr. ADAMS' theory on this subject, and he seems to have adopted it in order to designate a tumor whose external appearance is very different from the one just described. This tumor is prominent; its base or connexion with the side is not its greatest diameter; it is of a squared or angular form; the nipple stands prominent, the veins are of great size, and to a late state the tumor is loose from the pectoral muscles; it is not equally soft or elastic; and it is easier to turn it off from its connexion than to dissect out a common cancer. When cut into, it is composed of clusters of lesser tumors: these possess the common carcinomatous appearance; in the interstices of the tubercles large bags, or cells, are found, of a yellowish colour; the larger ones containing a dark fluid, like blood or bile. It is from the development of one or two of these cells, that the square and prominent form of this tumor is owing. In this particular tumor, the glands of the axilla remain a longer time than usual free from disease.

Mr. Bell next notices what he calls *the acute carcinomatous tumor of the mammæ,* principally remarkable for the rapidity of its progress.

*Cancer commencing in the Areola* is a rare disease, beginning in the glands of the areola, which become tuberculated and ulcerate; the breast appearing more round and elastic than the healthy one. It needs only to be added, that the fungous excrescence which rises from the surface may be repeatedly destroyed and reproduced, until at length the constitution becomes disturbed, and the patient sinks.

*The acute Fungous Tumor of the Mammæ.*—This variety runs its course with rapidity. The tumor occupies the whole gland; it appears as if it would point in two or three places; the colour is intensely red, and the surface granular: it does not, however, contain fluid; but a fibrous, soft fungus is disclosed, which bleeds sometimes profusely, and the patient dies hectic.

Two plates are added, to illustrate the internal structure of cancer. The first shows one of the diverging filaments longer than the rest, which extends to the utmost margin, and then appears abruptly cut off. The patient, whose breast is here represented, died, two years after the operation, of inflammation of the chest, and at the time of her death there was a hard tubercle on the skin of the breast, inclining to ulcerate; which Mr. Bell justly thinks to have been a scion or budding, probably, from the very filament above mentioned. Of the second plate we can give no idea by words. It is to be observed, that the third of Mr. Bell's drawings, which he describes, is not engraved in this volume.

*Dilator for the Female Urethra.*—This is a short paper from Sir Astley Cooper, giving an account of two operations, in which a dilator, suggested by Mr. Weis, of the Strand, was successfully employed. It consists of something like the blades of a straight forceps, which are introduced into the urethra closed. They are hollowed, and between them is placed a stile, which, by means of a screw in the handle, can be drawn back upon the inclined plane of the blades, and consequently made to force them open. The possibility of dilating the female urethra to a considerable extent, by means of sponge tent, was sufficiently established in former papers, by Sir A. Cooper, Mr. Thomas, and Mr. Travers; but the first of these gentlemen, having observed the inconveniences attending this method, was led to the adoption of the means now described. A lady had a stone in the bladder: the dilator was introduced at eight in the morning, and removed at four o'clock in the afternoon, when the passage was sufficiently dilated to admit the finger. A forceps was then passed into the bladder, and the stone extracted; but, being soft, its outer shell separated, which rendered it necessary to repeat the introduction of a flat forceps, in order to extract the fragments. This patient did well, suffering but little from the operation, and never having lost the power of retaining her urine. It is rather singular, however, that we are not told the size of the stone, nor do we get any hint by which we can conjecture to what extent the urethra was dilated; except, indeed, that it allowed the Baronet's finger to pass. In this instance it is to be observed that the dilatation was effected gradually, and thus far the operation resembled that of the tent sponge; in the next this was not the case. A lady had been in the habit of introducing a catheter for herself; on one of these occasions it broke, and the broken portion remained in the bladder. The dilator was retained for two minutes only, when it admitted the finger, and the foreign body was extracted by the forceps. The urine, in this case, passed involuntarily, until the next return of the catamenia: we are not told what period intervened before this took place. Sir Astley is of opinion that, if the stone be small, the dilatation may be accomplished in a few minutes; but, if large, it should be done gradually, "from day to day, till the greatest degree of extension is accomplished."

In the form of appendix to this paper, two cases are given of considerable interest. The first is an account of the extraction of a female catheter, which had slipped into the bladder by accident, and remained there nearly three weeks. The surgeon, Mr. Thomas Chapman, introduced a common forceps, then his little finger, and subsequently his fore-finger, by means of which, conjointly with the other hand applied externally, he guided the instrument to the orifice of the urethra. This operation was performed under very disadvantageous circumstances, the lady and her friends being kept in ignorance of the accident. In the second case, a stone was extracted by dilatation; and we are told that another would have been so likewise, had the lady remained quiet. The extraction was effected by touching "the orifice of the meatus with the point of a lancet."

*Tumor removed from the Neck.*—This paper concludes the volume, and is communicated by Mr. VINCENT, of St. Bartholomew's Hospital. A boy, aged six years, had a tumor which occupied " the whole of the right side of the neck, extending from the ear to the clavicle, and laterally from the edge of the trapezius to the trachea, projecting in a proportionate degree beyond the natural contour of the neck." Two semilunar incisions were made from the ear to the clavicle; the tumor was lobulated, and a large mass removed by cutting through the membranous septa; the remaining portions (one of which passed behind the internal jugular vein, and another lay in contact with the pleura,) were cautiously drawn out from their situations, and removed. There was very little bleeding; but the accessory nerve, and some branches of the cervical plexus, were divided. The boy recovered at the time, but shortly after died of disease of the lungs, which supervened upon measles: indeed, he seems to have had cough and difficulty of breathing from about ten days after the operation was performed. On examination after death, the right lung was found inflamed, and there were partial adhesions of the pleura; but the most remarkable circumstance is, that the left lobe of the lungs was completely destroyed, pus occupying its place. The fact that collections of matter are frequently found in the chest after great operations, appears to us not to have received the notice it deserves. In this instance the boy had an attack of erysipelas; and we have been informed, by one of the surgeons in charge of the military hospital at Chatham, that he found abscesses in the lungs of six or seven soldiers who died of that disease about two years ago, when it prevailed so extensively.

---

### DIVISION II.

### FOREIGN.

ART. III.—*Memoire Physiologique sur les Maladies Purulentes et Putrides sur la Vaccine, &c.* Par B. GASPARD, D.M.—(Journale de Physiologie.)

ART. IV.—*Recherches, Physiologiques et Medicales, sur l'Acetate de Plomb.* Par B. GASPARD, D.M.—(Journale de Physiologie.)

[Continued from page 79.]

IT seems scarcely credible that, in the present day, argument should be required to prove that lead is capable of producing deleterious effects upon the animal economy; a fact with which every workman in this metal is fully acquainted; yet a French physician, to whom Dr. GASPARD refers, maintains the innocuousness of the acetas plumbi,—recommends it in the daily dose of *twelve grains*,—and avers that the colic "dite de plomb" is improperly ascribed to lead. Notwithstanding the bold assertion of this writer, we have too good an opinion of the readers of our Journal to waste their time by an attempt to prove the absurdity of the latter statement. It is opposed to the observation of most practitioners, refuted by an abundance of recorded cases, and known to be erroneous by every child in the vicinity of a white-lead manufactory. Even animals, who are kept near the pans in which the sugar-of-lead is made, are destroyed. M. Merat speaks of eighteen dogs who perished from this circumstance: at the end of eight or ten days, they lost their appetite, became dull and costive, blood was evacuated by vomiting, and also with the fæces and urine; and they died after suffering constant agony.

Dr. Gaspard here supplies us with the details of some experiments, the first of which were made by him in the year 1816, to determine the effects of lead on the animal economy. They are deserving of notice, as demonstrating the effect of the direct introduction of this substance into the veins.

*Exp.* 1.—Dr. Gaspard injected, into the jugular vein of a middle-sized bitch, two grains of the acetate of lead, dissolved in an ounce of distilled water, which caused momentary expressions of pain. During the first three days, the animal was slightly indisposed, his appetite being diminished, and he had slight fever and thirst. On the fourth day, he became evidently ill, with marked fever, frequent pulse, no appetite, intense thirst, and dry nostrils. These symptoms increased on the following day, with debility and wasting. Moreover, on the sixth day, the urine was of a dark-red colour, like putrid blood; the dog frequently moaned, and died on the seventh. During these seven days, the animal voided fæces once only.

On the opening of the body, the lungs were somewhat inflamed, or ecchymosed in some places by patches or spots. The stomach was sound; but the small intestines were affected, especially in their muscular coat, where they were ecchymosed, gorged, and indurated. They were marked by peculiar inflammatory and gangrenous appearances, presenting here and there livid spots and vesicles filled with black and liquid blood. The mucous and serous membranes were almost healthy, but the interior of the intestinal tube was full of dirty mucous matters. The large intestines deviated but little from a healthy state, and contained pultaceous, sanguinolent, and fœtid feculent matter. The bladder contained a thick, greenish-brown liquid, similar to urine in which cow-dung had been mixed up.

*Exp.* 2.—The author put healthy young frogs, newly born, in water, containing acetate of lead in the proportion of half a grain to an ounce: they swam about for some time with vigour enough; but at length they remained at the bottom of the vessel, agitating their tails, and died in less than an hour.

*Exp.* 3.—Two grains of acetate of lead, dissolved in warm water, were injected into the cellular membrane upon the chest of a cat, which had been previously inflated: the animal immediately manifested pain by reiterated mewings and agitation; afterwards dyspnœa, anorexia, painful locomotion; a hard, painful, and inflammatory tumor of the injected cellular tissue, followed by suppuration and sloughing of the tissue on the tenth day. He was quite recovered at the end of January.

*Exp.* 4.—A grain of the acetate of lead, dissolved in an ounce and a half of distilled water, were injected into the jugular vein of a large bitch. In the course of the day, she manifested the same slight indisposition as in the animal subjected to the first experiment, and almost immediately evacuated her urine and fæces; but on the following day the indisposition increased, with great thirst, refusal of food, dry nostrils, depression of strength, and slight fever. He then introduced into the vein, anew, an ounce of distilled water, holding in solution another grain of sugar-of-lead. This second injection was followed by a new evacuation of fæces. During the succeeding days, the symptoms continued. After the third day, the animal cried frequently. On the fourth, she discharged from the bowels pultaceous, very fœtid, mucous, sanguine and black matter, as in scorbutic or gangrenous dysentery, with frequent tenesmus. The urine was small in quantity, but natural.

On the fifth day, she was evidently worse: the alvine excretions were very frequent, and sometimes consisted solely of black blood; the emaciation was great; locomotion vacillating, with weakness of the hinder extremities; frequent cough; vomiting; cries; finally, convulsive symptoms, and death.

On examination of the body, the lungs were found studded with livid spots; the small intestines showed similar spots, in great number, in their muscular and mucous tissues, but were otherwise healthy; the large intestines were a little thickened, without decided inflammation, but covered internally with black, putrid, mucous blood, similar to that which was voided during life: the other organs were sound.

*Exp.* 5.—Nine grains of acetate of lead, dissolved in an ounce and a half of spring water, were injected into the jugular of another dog, of middle size. The animal was agitated, and apparently in pain. On the following day, he was indisposed, with slight dyspnœa; belly was painful on pressure; the appetite was diminished, and he was thirsty: nevertheless the fever was moderate; but the nostrils were dry and inflamed, with frequent sneezings. On the fifth day, almost all these symptoms were calmed or dissipated; the appetite returned, and the animal appeared likely to recover. The author considered that the salt had been decomposed by the spring water, and consequently deprived of almost all its deleterious qualities: the experiment was, therefore, repeated on the 23d of April, by injecting into the vein an ounce and a half of distilled water *saturated* with acetate of lead, although very limpid. Immediately afterwards, the dog lost all appetite; had violent and repeated vomitings, four times in less than an hour. Soon afterwards, had an evacuation of fecal matters, followed by dysenteric tenesmus, excretion of blood by the anus, with great pain, prolapsus recti, &c. The animal had also dyspnœa, plaintive breathing, and fever; his chest and belly were painful on pressure. He became very seriously ill, lying, without strength, on his left side. Four hours after this second injection, he suddenly sent forth great cries, evacuated liquid and very fœtid stools; was seized with convulsive movements of the limbs and trunk; extreme agitation; singultuous respiration, with startings of the tendons; vomitings; and convulsive efforts, soon followed by death.

On opening the body, the lungs were found gorged and inflamed, although flaccid; they were studded with a number of dark patches from effused blood, and depending upon a particular inflammation. The mucous membrane of the intestines in the vicinity of the inflammation was red, and of the colour of wine-dregs. The gall-bladder was filled with black and very thick bile. The colon was inflamed and ecchymosed in its cul de sac, with effusion of blood between its membranes.

The conclusions to be drawn from the preceding experiments are too obvious to require mention. The author suspects that, where lead has been administered in large doses without deleterious effects, it must have been decomposed by the admixture of other medicines.

The author refers to an experiment of Brunner, who destroyed a dog by making him swallow litharge dissolved in vinegar; and of Hellefield, who witnessed inflammation of the stomach in a dog from the sugar of lead. Spröegel, in 1753, injected the acetate of lead into the veins of dogs, with results similar to those obtained by the author.

---

Art. V.—*Commentario Clinico per la Cura dell' Idrofobia.* Del Cavaliere Valeriano Luighi Brera.—(Memoria della Società Italiana delle Scienze residente in Modena. Tom. xviii. parte fisica.)

[Concluded from page 82.]

The subjects of the preceding narrative continued in health down to the time of writing the memoir, which must have been upwards of six years. It may, possibly, be objected, that they might not have been attacked by hydrophobia had they been left to themselves; and the possibility of this occurrence is still further strengthened by the fact, that, of a given number of persons bitten by a rabid animal, a certain proportion generally escape; yet, if we take into consideration that part of the account which we have marked by italics, we shall have some difficulty in believing but that three, at least, would have been attacked, had they not been subjected to the vigorous treatment of the author: they had, in fact, some of its characteristic symptoms. It should also be noted that several of the fatal cases were submitted to prophylactic treatment of a different kind, and that the patients above mentioned were *alone* saved: if, therefore, their immunity be not owing to the remedies exhibited, it is the most singular coincidence we ever met with. That the reader may be the better enabled to judge for himself, we subjoin a brief account of the other cases.

1. Chizzoli Pietro, fourteen years of age, had a laceration in the forehead, laying bare the frontal bone; also another extensive wound in the angle of the lower jaw. The hemorrhage was profuse; he had convulsions, and his strength was greatly depressed.

*Symptoms.*—The convulsions had ceased, and the wounds were healed, when he became hydrophobic on the 24th of December, with horror at the sight of water and light.

*Remedies,* in the space of twenty-six days, were 78 grains of opium, 14 scruples of musk, 215 drops of caustic alkali in solution, externally; an ounce and five drachms of mercurial liniment. The effect of these were night sweats, increased flow of urine, salivation, without pain and heat of the mouth.

*Event.*—Died tranquilly on the 26th of December.

2. Limetti Michele, aged twenty-seven, of robust habit. His wounds were cutaneous, and but slight, in the right angle of the mouth and ear of the same side.

On the 16th of November, they were already healed; he became hydrophobic on the 17th. The symptoms were melancholy, fever, dysphagia, aversion of water, flow of saliva, convulsions, and stupor.

*Event.*—Died comatose on the 19th of November.

3. Cremonese Antonio, aged thirteen. Deep lacerations upon the head and neck, left carotid was opened; the hands and legs were also bitten. Hemorrhage was profuse, which left him half dead, with convulsions, and threatening of tetanus; coma succeeded to the convulsions, after which he recovered. On the 18th of November, hydrophobic fever appeared, with type of a simple tertian, (to use the author's expression.) On the 20th, the hydrophobia was more manifest: he had night sweats, increased flow of urine, spasms, difficulty in drinking water, but *facility in drinking wine*, with ejection of frothy saliva, and disposition to bite the bystanders.

*Remedies*, in four days, were wine, China, serpentaria, four scruples of musk, two scruples of camphor, eight scruples of the liq. c. c. succinatus, and twelve grains of opium.

*Event.*—Died, in great anxiety, on the 24th of November.

The 4th, 5th, and 6th, are those of Peverari Girolani, Giroletti Agostino, and Frassini Grambattisto, above related; the latter, by mistake, under the name of Agostino.

7. Baita Agostina, twenty-two years of age. The integuments at the top of the head were extensively lacerated; the pericranium was laid bare and torn; he suffered great loss of blood, with exhaustion of strength, convulsive tremors, and continual restlessness. The wounds were healed at the end of December, when threatenings of hydrophobia appeared in the course of the prophylactic treatment, which were subdued. He was at length attacked by the disease on the 13th of May, and died convulsed on the 16th.

*Remedies*, administered in forty-seven days, were 148 grains of calomel, 152 of opium; externally, mercurial liniment, by friction, three ounces and a half, and nineteen baths; which produced night sweats, increased flow of urine, and long continued salivation.

8th. Ghilardi Luigi, nineteen years of age. Laceration in both hands, in the thigh, and left foot, with considerable loss of blood and convulsions. His wounds were healed on the 17th of November; he became hydrophobic on the 21st of December.

*Remedies*, in thirty-three days, were nineteen ounces and a half of diluted sulphuric acid; externally, mercurial ointment, by friction, four ounces and a half; he went into a warm bath thirteen times. This treatment produced copious sweats, abundance of urine, and continual salivation.

*Event.*—Died, convulsed, on the 22d of December.

9th. Comanduli Luigi, aged twenty-six, slightly bitten in the right shoulder. On the 9th of November the wounds were closed; the cicatrices became painful. He remained two years without attack, when at length he became debilitated, delirious, and rabid on the 12th of April, 1810, *without aversion to water*.

*Event.*—Died, convulsed, on the 14th of April of the year 1810.

*Remedies*, in forty-three days, twenty-one ounces and a half of sulphuric acid; mercurial liniment, by friction, two ounces and a half; went into a warm bath twelve times. His wounds were cauterized on the 1st of November. He was under salivation during the whole of the treatment, with night sweats and increased flow of urine. His cicatrices were painful.

10th. Zucchetti Catterina, aged eleven, bitten in six places in the face and neck, with fracture of the lower jaw; was not submitted to any treatment. Hydrophobia supervened on the 14th of November, with aversion to water and to the light reflected from a looking-glass. Died on the 15th of November.

11th. Zucchetti Francesca, aged forty, mother of the preceding; a robust woman; slightly bitten in the right arm. Wounds were healed on the 15th of November; on the 29th she became hydrophobic, although at the time in a state of profuse salivation, with inflamed gums and fauces. Her aversion to water, and to the light of a candle, was insuperable. She was greatly oppressed, had vomiting, cold sweats, lividness of the fauces, and ejection of frothy saliva.

*Remedies*, in six days, a stimulating antispasmodic mixture; calomel, forty-eight grains. Externally, cauterization of the wounds; mercurial liniment, by friction, six drachms.

*Event.*—Died, with stertorous breathing, on the 1st day of December.

12th. Zucchetti Maria Catterina, aged fifty-four, sister of the following, had laceration of the teguments of the throat and lower lip on the right side. The wounds were cauterized, and kept in a state of suppuration till the 17th of November. On the 18th of December, hydrophobic symptoms appeared; and on the 20th of December she died.

*Remedies*, in thirteen days, a stimulating mixture; externally, an ounce and a half of mercurial liniment was consumed by friction, and produced enormous salivation, with inflammation of the tongue.

13th, is the case of Zucchetti Carlo Giuseppe, whose recovery has been before given.

The following are the inferences deduced by the author.

I. Among the bitten persons, the tenth was the first to perish from hydrophobia; after which the attack was in the following order,—the second, the third, the eleventh, the twelfth, the eighth, the first, the seventh, and the ninth. The seventh remained free from the disease more than ten months; the ninth, *five years and a half.*

II. Each of the individuals numbered 1, 3, 7, and 8, who died, lost a considerable quantity of blood from the bites.

III. In the persons numbered 2, 9, and 11, the integuments were scarcely injured; nevertheless they perished. In the 9th, the teeth of the wolf must have pierced a coarse cloth and other garments.

IV. The individuals 2 and 8 were robust persons, and were attacked before those who were debilitated by hemorrhagy at the time of the accident.

V. The wounds suppurated, naturally, a long time in the individuals 1, 3, 7, and 13; and in consequence of cauterization in 2, 9, 11, and 12; all of whom were the victims of hydrophobia. The wounds in those who escaped were not cauterized, and in a few days were cicatrized.

VI. Night sweats, abundant flow of urine, and salivation produced by mercury, were not critical phenomena, inasmuch as they were observed as well in those who died as in those who survived.

VII. Insuperable aversion to water, a phenomenon common to rabid persons, as was observed in No. 11, was not present in No. 9. It was also remarked in the survivors Nos. 4, 5, and 13. Aversion to light reflected from a looking-glass was experienced in No. 10; aversion to the light of a candle, and to the agitation of the air, was remarked in No. 11.

VIII. Nos. 3 and 5 swallowed water with difficulty, but wine with ease.

IX. Pains in the wounds and cicatrices were observed in the surviving persons, 4 and 13.

X. Dryness of the fauces, abundant and frothy saliva, vigilance, eyes more than usually fierce and shining, and melancholy, were observed both in the living and in the dead. In No. 12, the disease was fatal without ejection of frothy saliva.

XI. If extreme melancholy had the power of contributing to the attack of the disease, as maintained by Bosquillon, the individual, No. 13, who survived, would not have escaped; more especially as he evinced some symptoms.

XII. The disposition to bite the bystanders was observed only in the individual No. 3.

XIII. The paroxysms of hydrophobia were evidently intermittent in the fatal case No. 3.

XIV. Nos. 3, 7, 8, 9, and 10, died convulsed; Nos. 1 and 12 died tranquilly; Nos. 2 and 11 comatose.

XV. Lividity of the face and body were manifest after death in Nos. 8 and 9.

XVI. Among the phenomena observed in examination after death, an inflammation of the spinal marrow was observable in No. 10. The symptoms with which No. 9 was affected leaves reason to conjecture that an organic degeneration of the spinal marrow, from inflammation, would have been discovered on examination, had it been instituted.

XVII. In Nos. 1, 2, 3, 7, and 11, opium, either alone or combined with musk, cantharides, caustic alkali, mercury, camphor, ammonia, and succinat, were useless.

XVIII. Caustic alkali, in good doses, was administered in No. 8.

XIX. Diluted sulphuric acid, largely administered, was useless in the cases 8 and 9.

XX. To all the patients, excepting the child, mercurials were administered so as to produce salivation. In No. 7, this remedy seemed to have the effect of subduing some threatenings of hydrophobia, although, some months afterwards, the patient died of the disease. To the survivors 5, 6, and 13, a smaller dose of the mercurial was administered than to those who died.

XXI. All the patients used the warm bath.

XXII. One of the survivors, No. 4, at the time of receiving the bite, threw himself in a ditch full of cold water.

XXIII. A seton was applied to the survivor No. 13.

XXIV. Of twelve persons treated, only four survived, to whom the *atropa belladonna was administered in considerable doses.* The effects of the remedy were general debility, vertigo, obscurity of vision, and, finally, temporary blindness.

